# Tumor Burden Modifies the Prognostic Impact of Adequate Lymph Node Dissection in Patients Undergoing Curative-Intent Resection for Intrahepatic Cholangiocarcinoma

**DOI:** 10.1245/s10434-026-19995-2

**Published:** 2026-06-14

**Authors:** Jun Kawashima, Kota Sahara, Kizuki Yuza, Yutaka Endo, François Cauchy, Federico Aucejo, Hugo P. Marques, Rita Lopes, Andreia Rodriguea, Tom Hugh, Feng Shen, Shishir K. Maithel, Bas Groot Koerkamp, Irinel Popescu, Minoru Kitago, Matthew J. Weiss, Guillaume Martel, Carlo Pulitano, Luca Aldrighetti, George Poultsides, Andrea Ruzzente, Todd W. Bauer, Ana Gleisner, Yuki Homma, Itaru Endo, Timothy M. Pawlik

**Affiliations:** 1 Department of Hepatobiliary and Pancreatic Surgery, Yokohama City University Graduate School of Medicine, Yokohama, Japan; 2 Department of Surgery, The Ohio State University Wexner Medical Center and James Comprehensive Cancer Center, Columbus, OH USA; 3 Department of Transplant Surgery, University of Rochester Medical Center, Rochester, NY USA; 4 Department of HPB Surgery and Liver Transplantation, Beaujon Hospital, Clichy, France; 5 Department of Hepato-pancreato-biliary & Liver Transplant Surgery, Digestive Diseases and Surgery Institute, Cleveland Clinic Foundation, Cleveland, OH USA; 6 Department of Surgery, Curry Cabral Hospital, Lisbon, Portugal; 7 Department of Surgery, The University of Sydney, Sydney, NSW Australia; 8 Department of Surgery, Eastern Hepatobiliary Surgery Hospital, Shanghai, China; 9 Division of Surgical Oncology, Winship Cancer Institution, Emory University, Atlanta, GA USA; 10 Department of Surgery, Erasmus University Medical Centre, Rotterdam, The Netherlands; 11 Department of Surgery, Fundeni Clinical Institute, Bucharest, Romania; 12 Department of Surgery, Keio University, Tokyo, Japan; 13 Department of Surgery, Cancer Institute, Northwell Health, New Hyde Park, NY USA; 14 Department of Surgery, University of Ottawa, Ottawa, ON Canada; 15 Department of Surgery, Royal Prince Alfred Hospital, Camperdown, NSW Australia; 16 Department of Surgery, San Raffaele Hospital, Milan, Italy; 17 Department of Surgery, Stanford University, Stanford, CA USA; 18 Division of General and Hepatobiliary Surgery, University of Verona, Verona, Italy; 19 Department of Surgery, University of Virginia, Charlottesville, VA USA; 20 Department of Surgery, University of Colorado Denver, Denver, CO USA

## Abstract

**Introduction:**

The oncologic impact of lymph node dissection (LND) for intrahepatic cholangiocarcinoma (iCCA) remains unclear. We hypothesized that the prognostic relevance of LND may vary according to tumor burden. Therefore, this study sought to evaluate the interaction between tumor burden and adequate LND among patients who underwent curative-intent resection for iCCA.

**Methods:**

Patients who underwent curative-intent liver resection for iCCA were identified from a large international multi-institutional database. Overall survival (OS) was evaluated using multivariable Cox regression models that included an interaction term between tumor burden score (TBS) and adequate LND.

**Results:**

Among 1,558 patients, 872 (56.0%) underwent LND and 322 (20.7%) underwent adequate LND, defined as retrieval of at least six lymph nodes. The median TBS was 6.1 (interquartile range [IQR] 4.1–8.6). On multivariable Cox regression analysis, a significant interaction was observed between TBS and adequate LND (hazard ratio [HR] 0.91, 95% confidence interval [CI] 0.87–0.95, *p* < 0.001). Among 542 (34.8%) patients with TBS < 5.0, adjusted OS did not differ according to adequate LND status (HR 1.23, 95% CI 0.86–1.76, *p* = 0.265). In contrast, among 1,016 (65.2%) patients with TBS ≥ 5.0, adequate LND was associated with improved adjusted OS (HR 0.65, 95% CI 0.51–0.82, *p* < 0.001). Similar findings were observed for recurrence-free survival (RFS).

**Conclusions:**

The prognostic relevance of adequate LND in patients undergoing curative-intent resection for iCCA appears to vary according to tumor burden. Adequate LND was associated with improved OS and RFS among patients with high TBS, but not among those with low TBS.

**Supplementary Information:**

The online version contains supplementary material available at 10.1245/s10434-026-19995-2.

Intrahepatic cholangiocarcinoma (iCCA) is the second most common primary liver malignancy after hepatocellular carcinoma, and its incidence has steadily increased worldwide over the past several decades.^1,2^ Surgical resection remains the cornerstone of curative-intent treatment for patients with resectable iCCA.^3^ Nevertheless, long-term outcomes remain unsatisfactory with reported 5-year overall survival (OS) ranging from 25 to 40%.^1^ Given these unsatisfactory outcomes, optimizing surgical management has become a major clinical priority for patients undergoing resection for iCCA.

Among the clinicopathologic factors associated with prognosis, lymph node metastasis has consistently been identified as one of the most important determinants of survival in iCCA.^3^ Accordingly, the eighth edition of the American Joint Committee on Cancer (AJCC) staging system recommends evaluation of at least six lymph nodes for accurate nodal staging.^4^ Despite the recognized prognostic importance of nodal disease, the oncologic value of regional lymph node dissection (LND) itself remains controversial.^5^ Some studies have suggested that adequate LND may improve survival, whereas others have failed to demonstrate a clear oncologic benefit.^6–8^ One possible explanation for these inconsistent findings is that the impact of LND may not be uniform across all patients. Adequate LND may improve the accuracy of nodal staging, thereby reducing stage migration and providing more complete pathologic nodal information for postoperative prognostic assessment. In addition, LND may also provide a therapeutic benefit through removal of regional lymph node metastatic disease. If LND were to have a therapeutic benefit, the prognostic significance of adequate LND may depend, at least in part, on underlying tumor biology and burden.

Tumor burden, as reflected by tumor size and number, is a well-established predictor of survival in iCCA and, importantly, can be assessed preoperatively.^8–10^ We hypothesized that the prognostic relevance of adequate LND may vary according to tumor burden. In turn, the objective of the current study was to evaluate the interaction between tumor burden and adequate LND using a large international multi-institutional database of patients who underwent curative-intent resection for iCCA.

## Methods

### Data Source and Patient Selection

Patients who underwent curative-intent liver resection for iCCA between 2000 and 2024 were identified from the International Intrahepatic Cholangiocarcinoma Study Group database.^11^ Patients with extrahepatic metastatic disease or an R2 resection were excluded. In addition, patients with missing data on tumor size, tumor number, LND status, or the total number of lymph nodes examined (TLNE) were excluded from the analysis. The study protocol was approved by the institutional review board at each participating institution.

### Variables and Outcomes

Collected variables included demographic and clinicopathologic characteristics, such as age, sex, American Society of Anesthesiologists (ASA) classification, year of surgery (2000–2010 vs. 2011–2024), receipt of neoadjuvant chemotherapy (NAC), presence of jaundice, presence of cirrhosis, type of resection (major hepatectomy: ≥3 segments vs. minor hepatectomy: ≤2 segments),^12^ use of minimally invasive surgery (MIS), preoperative lymph node status (N0, N1), LND, TLNE, tumor size (cm), tumor number, tumor burden score (TBS),^13^ T category (AJCC 8th edition),^4^ nodal status (N0, N1, Nx), R1 resection, major vascular invasion, microvascular invasion (MVI), morphological subtype (MF, mass-forming; IG, intraductal growth; PI, periductal infiltrating; MF+PI, periductal infiltrating plus mass-forming), tumor grade (well, moderate, poor, undifferentiated), perineural invasion (PNI), postoperative severe complications, and receipt of adjuvant chemotherapy. Adequate LND was defined as LND with TLNE of 6 or more.^4^ TBS, a composite index of tumor burden that incorporates maximum tumor size and number, was computed based on the formula: TBS^2^ = (maximum diameter)^2^ + (number of tumor)^2^. R1 resection was defined as a surgical margin width of < 1 mm on permanent histologic examination, whereas R0 resection was defined as ≥1 mm.^14^ Severity of postoperative complications was classified according to the Clavien-Dindo grading system with severe complications defined as grade ≥ III.^15^

The primary outcome was OS, defined as the time from resection to death from any cause or last follow-up. The secondary outcome was recurrence-free survival (RFS), defined as the interval from the date of liver resection to the date of recurrence either pathologically confirmed or radiographically suspected on follow-up imaging.

### Statistical Analysis

Continuous variables were summarized as median values with interquartile ranges (IQR) and compared using the Mann–Whitney *U* or Kruskal–Wallis tests, as appropriate. Categorical variables were reported as frequencies and percentages and compared using the χ^2^ or Fisher’s exact test. Missing data were handled using multiple imputation with chained equations (MICE).^16^ Survival curves were estimated using the Kaplan–Meier method and compared with the log-rank test.

Univariable Cox proportional hazards regression analyses were performed to identify factors associated with OS. Variables with a *p* value < 0.1 on univariable analysis, as well as clinically relevant covariates, were entered into multivariable Cox proportional hazards regression models. To evaluate whether the prognostic impact of adequate LND differed according to tumor burden, an interaction term between adequate LND and TBS was included in the primary multivariable model. Covariates included in the primary interaction model were ASA classification, jaundice, preoperative lymph node status, TBS, adequate LND, and major vascular invasion.

To further illustrate the interaction between tumor burden and adequate LND, the conditional hazard ratio of adequate LND across the continuous range of TBS was estimated from the interaction model and plotted with 95% confidence intervals. For stratified survival analyses, patients were categorized according to TBS <5 versus ≥5. This threshold was informed by the conditional hazard ratio plot derived from the interaction model, in which the estimated hazard ratio for adequate LND crossed 1 at approximately TBS = 4.68; for ease of interpretation, this value was rounded to 5. Adjusted survival curves for OS and RFS were then generated after stratifying patients according to TBS (<5 vs. ≥5) and adequate LND status. These adjusted curves were estimated by standardization based on multivariable Cox models, including adequate LND, ASA classification, jaundice, preoperative lymph node status, major vascular invasion, and TBS.

To examine the joint association of tumor burden and extent of nodal evaluation with prognosis, contour plots were generated using multivariable Cox models with restricted cubic splines for TBS and TLNE. Standardized 5-year OS probability was estimated across the continuum of TBS and TLNE using g-computation while averaging over the observed distribution of other covariates.

A prespecified subgroup analysis was performed among patients with clinically node-negative disease (cN0; preoperative N0) to assess whether the association between adequate LND and outcomes according to tumor burden persisted in the absence of preoperatively suspected nodal disease. Within the cN0 subgroup, adjusted OS and RFS curves were similarly constructed after stratification by TBS (<5 vs. ≥5) and adequate LND status. In the entire cohort, an exploratory analysis was performed to examine the interaction between any LND (yes vs. no) and TBS using a multivariable Cox model adjusted for ASA classification, jaundice, preoperative lymph node status, TBS, LND status, and major vascular invasion. In the entire cohort, a sensitivity analysis was also performed in which the primary interaction model was further adjusted for postoperative pathologic variables, including adjuvant chemotherapy, resection margin status, morphologic subtype, tumor grade, PNI, MVI, nodal status, and T category.

To assess potential institutional bias related to center-level LND practice patterns, the institution-level adequate LND rate was calculated for each participating center. Institutions were then ranked according to the adequate LND rate and categorized into high- and low-adequate-LND-rate centers using the median value to divide the categories. OS was compared between high- and low-adequate-LND-rate centers using Kaplan–Meier analysis and the log-rank test. Furthermore, to assess postoperative prognostic stratification by pathologic nodal status, Harrell’s C-index for pathologic nodal status was calculated for OS and RFS according to the adequacy of LND in the entire cohort. For this analysis, Nx was treated as N0.

All statistical analyses were performed using R software (version 4.4.2; R Foundation for Statistical Computing, Vienna, Austria). All tests were two-sided, and *p* values < 0.05 were considered statistically significant.

## Results

### Patient Characteristics

Among the 1,558 patients who met inclusion criteria, 844 (54.2%) were male, and median age was 62 years (IQR, 53–70). A total of 667 (42.8%) patients had an ASA class >2, 121 (7.8%) presented with jaundice, and 118 (7.6%) received NAC. Overall, 872 (56.0%) patients underwent LND, and 322 (20.7%) underwent adequate LND, defined as retrieval of at least six lymph nodes. The median TLNE was 1 (IQR, 0–1). Among 69 patients who underwent MIS, any LND was performed in 23 patients (33.3%), whereas adequate LND was achieved in nine patients (13%). The median tumor size was 6.0 cm (IQR, 4.0–8.0), and 234 (15%) patients had multiple lesions. The median TBS was 6.1 (IQR, 4.1–8.6). On pathologic assessment, 789 (50.6%) patients had T1 disease and 327 (21%) had nodal metastasis (N1). R1 resection occurred in 254 (16.3%) patients, while major vascular invasion was present in 228 (14.6%) individuals. In addition, MVI, PI/MF+PI type, poorly or undifferentiated tumors, and PNI were present in 482 (30.9%), 226 (14.5%), 278 (17.8%), and 340 (21.8%) patients, respectively. Overall, 326 (20.9%) patients experienced a postoperative severe complication, and 460 (29.5%) received adjuvant chemotherapy (Table 1).

**Table 1:** Clinicopathological characteristics of the analytic cohort

Characteristics	All patients	TLNE < 6	TLNE ≥ 6	*p*
	*n* = 1558	*n* = 1236	*n* = 322	
Age, years, median (IQR)	62 [53, 70]	61 [52, 69]	65 [57, 72]	<0.001
Sex, male	844 (54.2)	697 (56.4)	147 (45.7)	0.001
ASA classification >2	667 (42.8)	543 (43.9)	124 (38.5)	0.091
Year of surgery, 2011–2024	990 (63.5)	729 (59)	261 (81.1)	<0.001
Neoadjuvant chemotherapy	118 (7.6)	68 (5.5)	50 (15.5)	<0.001
Jaundice	121 (7.8)	76 (6.1)	45 (14)	<0.001
Cirrhosis	187 (12)	177 (14.3)	10 (3.1)	<0.001
Major hepatectomy	948 (60.8)	683 (55.3)	265 (82.3)	<0.001
Minimally invasive surgery	69 (4.4)	60 (4.9)	9 (2.8)	0.148
Preoperative LN assessment, positive	349 (22.4)	233 (18.9)	116 (36)	<0.001
Lymphadenectomy	872 (56)	550 (44.5)	322 (100)	<0.001
TLNE, median (IQR)	1 [0, 4]	0 [0, 2]	9 [7, 13]	<0.001
Tumor size, median (IQR)	6 [4.0, 8.0]	5.8 [4.0, 8.0]	6 [4.0, 9.0]	0.039
Multiple lesions	234 (15)	187 (15.1)	47 (14.6)	0.88
Tumor burden score	6.1 [4.1, 8.6]	6.1 [4.1, 8.3]	6.4 [4.1, 9.2]	0.045
Pathological T category				<0.001
T1	789 (50.6)	686 (55.5)	103 (32)	
T2	286 (18.4)	206 (16.7)	80 (24.8)	
T3	336 (21.6)	229 (18.5)	107 (33.2)	
T4	147 (9.4)	115 (9.3)	32 (9.9)	
Pathological N category				<0.001
N0	545 (35)	384 (31.1)	161 (50)	
N1	327 (21)	166 (13.4)	161 (50)	
Nx	686 (44)	686 (55.5)	0 (0)	
Surgical margin, R1	254 (16.3)	183 (14.8)	71 (22)	0.002
Major vascular invasion	228 (14.6)	126 (10.2)	102 (31.7)	<0.001
Microvascular invasion	482 (30.9)	315 (25.5)	167 (51.9)	<0.001
Morphologic type, PI/MF+PI	226 (14.5)	148 (12)	78 (24.2)	<0.001
Grade, poor/undifferentiated	278 (17.8)	209 (16.9)	69 (21.4)	0.071
Perineural invasion	340 (21.8)	194 (15.7)	146 (45.3)	<0.001
Severe complication	326 (20.9)	229 (18.5)	97 (30.1)	<0.001
Adjuvant chemotherapy	460 (29.5)	306 (24.8)	154 (47.8)	<0.001

Data are numbers with percentages in parentheses or medians with interquartile ranges in brackets unless otherwise indicated. *ASA* American society of Anesthesiologists; *LN* lymph node; *TLNE* the total number of lymph nodes evaluated; *PI/MF+PI* periductal infiltrating/ mass forming plus periductal infiltrating

Adequate LND was performed in 322 (20.7%) patients. Compared with patients who did not undergo adequate LND, individuals who underwent adequate LND were more likely to have received NAC (15.5% vs. 5.5%, *p* < 0.001), present with jaundice (14.0% vs. 6.1%, *p* < 0.001), be classified as preoperative N1 (36% vs. 18.9%, *p* < 0.001), and undergo major hepatectomy (82.3% vs. 55.3%, *p* < 0.001). In addition, patients in the adequate LND group had a slightly larger tumor size (median, 6.0 [IQR, 4.0–9.0] vs. 5.8 [IQR, 4.0–8.0], *p* = 0.039) and higher TBS (median, 6.4 [IQR, 4.1–9.2] vs. 6.1 [IQR, 4.1–8.3], *p* = 0.045). On pathologic assessment, patients who underwent adequate LND were more likely to have T3/4 disease (56.3% vs. 33.6%, *p* < 0.001), nodal metastasis (50% vs. 13.4%, *p* < 0.001), major vascular invasion (31.7% vs. 10.2%, *p* < 0.001), MVI (51.9% vs. 25.5%, *p* < 0.001), PI/MF+PI type (24.2% vs. 12.0%, *p* < 0.001), and PNI (45.3% vs. 15.7%, *p* < 0.001). Furthermore, patients who underwent adequate LND were more likely to experience a severe complication (30.1% vs. 18.5%, *p* < 0.001) and receive adjuvant chemotherapy (47.8% vs. 24.8%, *p* < 0.001) (Table 1).

### Interaction between Tumor Burden Score and the Prognostic Impact of Adequate LND

In the entire cohort, after a median follow-up of 21.9 months (IQR, 10.5–43.7), the estimated 5-year OS was 40.7% (95% CI, 37.7–44.0). On multivariable Cox regression analysis that included an interaction term between TBS and adequate LND, ASA class >2 (hazard ratio [HR] 1.16, 95% confidence interval [CI] 1.01–1.35, *p* = 0.040), jaundice (HR 1.48, 95% CI 1.17–1.87, *p* = 0.001), preoperative lymph node metastasis (HR 1.25, 95% CI 1.06–1.49, *p* = 0.009), and major vascular invasion (HR 1.56, 95% CI 1.28–1.91, *p* < 0.001) were independently associated with worse OS. Notably, a significant interaction was observed between TBS and adequate LND (HR 0.91, 95% CI 0.87–0.95, *p* < 0.001) (Table 2).

**Table 2:** Univariable and multivariable Cox regression analysis for overall survival

	Univariate analysis		Multivariate analysis	
Variables	HR (95% CI)	*p*	HR (95% CI)	*p*
Age	1.00 [1.00, 1.01]	0.263		
Sex, male				
Female	Ref			
Male	1.11 [0.96, 1.28]	0.165		
ASA classification >2				
Classification 1, 2	Ref		Ref	
Classification >2	1.20 [1.04, 1.38]	**0.014**	1.16 [1.01, 1.35]	**0.040**
Year of surgery, 2011–2024				
2000–2010	Ref			
2011–2024	1.03 [0.89, 1.19]	0.700		
Neoadjuvant chemotherapy				
No	Ref			
Yes	1.07 [0.80, 1.43]	0.649		
Cirrhosis				
No	Ref			
Yes	1.15 [0.94, 1.42]	0.181		
Jaundice				
No	Ref		Ref	
Yes	1.60 [1.27, 2.01]	**<0.001**	1.48 [1.17, 1.87]	**0.001**
Preoperative LN assessment				
Negative	Ref		Ref	
Positive	2.17 [1.76, 2.68]	**<0.001**	1.25 [1.06, 1.49]	**0.009**
Tumor burden score	1.08 [1.06, 1.10]	**<0.001**		
Major vascular invasion				
No	Ref		Ref	
Yes	1.64 [1.36, 1.98]	**<0.001**	1.56 [1.28, 1.91]	**<0.001**
Adequate LND				
TLNE < 6	Ref			
TLNE ≥ 6	0.96 [0.80, 1.15]	0.683		
TBS * Adequate LND			0.91 [0.87, 0.95]	**<0.001**

*ASA* American Society of Anesthesiologists; *LN* lymph node; *TLNE* the total number of lymph nodes evaluated; *LND* lymph node dissection; *TBS* tumor burden score. Bold font signify *p* value < 0.05

**Fig. 1:**
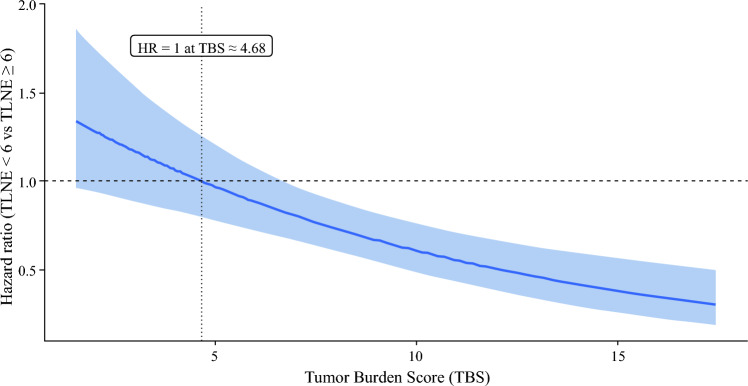
Conditional effect of adequate lymph node dissection across continuous tumor burden

Figure 1 illustrates the conditional effect of adequate LND across the continuous spectrum of TBS. As TBS increased, the estimated hazard ratio for adequate LND gradually decreased. The hazard ratio crossed 1 at approximately TBS = 4.68, suggesting that the prognostic association of adequate LND was limited among patients with lower tumor burden but became more apparent among individuals with higher tumor burden.

**Fig. 2:**
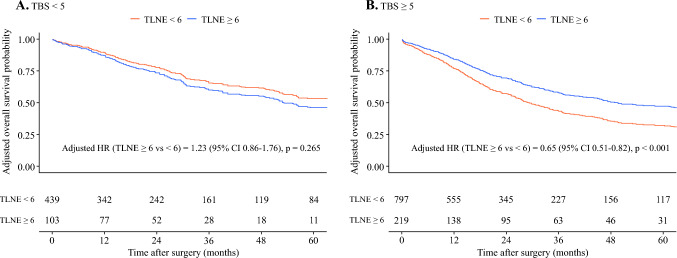
Adjusted overall survival by adequate lymph node dissection according to tumor burden score strata

**Fig. 3:**
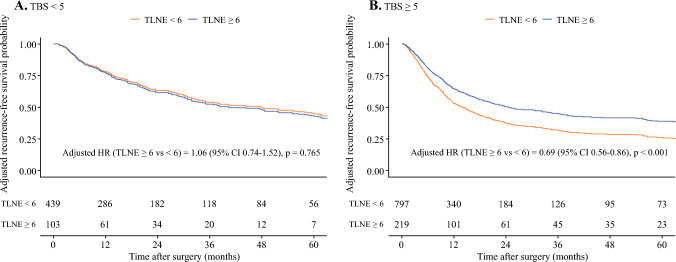
Adjusted recurrence-free survival by adequate lymph node dissection according to tumor burden score strata

Following stratification by TBS, among the 542 (34.8%) patients with TBS <5.0, adjusted OS did not differ according to adequate LND status (HR 1.23, 95% CI 0.86–1.76, *p* = 0.265) (Fig. 2A). Adjusted 5-year OS was 46.2% in the adequate LND group versus 53.1% in the nonadequate LND group. In contrast, among the 1,016 (65.2%) patients with TBS ≥5.0, adequate LND was associated with improved adjusted OS (HR 0.65, 95% CI 0.51–0.82, *p* < 0.001) (Fig. 2B), with an adjusted 5-year OS of 47.3% in the adequate LND group versus 32.2% in the nonadequate LND group. Similarly, among patients with TBS <5.0, adjusted RFS did not differ according to adequate LND status (HR 1.06, 95% CI 0.74–1.52, *p* = 0.765) (Fig. 3A). Adjusted 3-year RFS was 52.2% in the adequate LND group versus 54% in the nonadequate LND group. By contrast, among patients with TBS ≥5.0, adequate LND was associated with improved adjusted RFS (HR 0.69, 95% CI 0.56–0.86, *p* < 0.001) (Fig. 3B), with an adjusted 3-year RFS of 44.9% in the adequate LND group versus 32.0% in the non-adequate LND group.

**Fig. 4:**
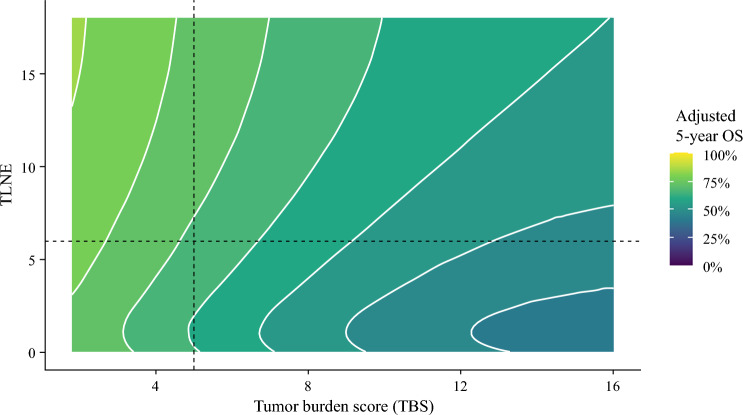
Adjusted 5-year overall survival across continuous tumor burden score and the total number of lymph nodes examined (TLNE)

Figure 4 demonstrates the joint association of TBS and TLNE with adjusted 5-year OS. In the lower TBS range, the contour lines were relatively vertical, indicating that prognosis varied mainly according to TBS. By contrast, in the higher TBS range, the contour lines became progressively more oblique, suggesting that the association between greater TLNE and prognosis became more apparent as tumor burden increased.

### Additional Analysis among Patients with Clinically Node-negative Disease

Among the 1,209 patients with clinically node-negative disease, 206 (17.0%) underwent adequate LND. Among the 441 patients with TBS <5.0, adjusted OS did not differ according to adequate LND status (HR 1.07, 95% CI 0.69–1.67, *p* = 0.760) (Supplementary Fig. 1A). Adjusted 5-year OS was 52.5% in the adequate LND group versus 54.7% in the nonadequate LND group. In contrast, among the 768 patients with TBS ≥5.0, adequate LND was associated with better adjusted OS (HR 0.71, 95% CI 0.54–0.94, *p* = 0.018) (Supplementary Fig. 1B), with an adjusted 5-year OS of 47.4% in the adequate LND group versus 35.6% in the nonadequate LND group. Similarly, among patients with TBS <5.0, adjusted RFS did not differ according to adequate LND status (HR 1.01, 95% CI 0.65–1.57, *p* = 0.961) (Supplementary Fig. 2A). Adjusted 3-year RFS was 54.7% in the adequate LND group versus 55.1% in the nonadequate LND group. By contrast, among patients with TBS ≥5.0, adequate LND was associated with better adjusted RFS (HR 0.64, 95% CI 0.48–0.85, *p* = 0.002) (Supplementary Fig. 2B), with an adjusted 3-year RFS of 50.4% in the adequate LND group versus 34.8% in the nonadequate LND group.

### Exploratory and Sensitivity Analyses

As an exploratory analysis, a multivariable Cox regression model incorporating an interaction term between any LND and TBS was constructed. In this model, ASA class >2 (HR 1.19, 95% CI 1.03–1.38, *p* = 0.017), jaundice (HR 1.51, 95% CI 1.19–1.92, *p* = 0.001), preoperative lymph node positivity (HR 1.23, 95% CI 1.03–1.46, *p* = 0.022), and major vascular invasion (HR 1.49, 95% CI 1.22–1.82, *p* < 0.001) were independently associated with worse OS. Notably, a significant interaction between TBS and LND was also observed (HR 0.90, 95% CI 0.86–0.94, *p* < 0.001) (Supplementary Table 1).

To further assess the robustness of the primary findings, a sensitivity analysis was performed by additionally adjusting for postoperative pathologic variables. In this model, ASA class >2 (HR 1.32, 95% CI 1.14–1.54, *p* < 0.001), jaundice (HR 1.42, 95% CI 1.11–1.81, *p* = 0.005), pathologic T3/4 disease (HR 1.22, 95% CI 1.03–1.45, *p* = 0.023), nodal metastasis (HR 2.19, 95% CI 1.81–2.65, *p* < 0.001), PI/MF+PI type (HR 1.34, 95% CI 1.09–1.64, *p* = 0.005), PNI (HR 1.23, 95% CI 1.01–1.50, *p* = 0.041), and receipt of adjuvant chemotherapy (HR 0.66, 95% CI 0.56–0.78, *p* < 0.001) remained independently associated with OS. Importantly, the interaction between TBS and adequate LND remained significant even after adjustment for postoperative pathologic variables (HR 0.90, 95% CI 0.86–0.94, *p* < 0.001) (Supplementary Table 2).

### Additional Institution-Level Sensitivity Analysis

To assess potential institutional bias, institutions were categorized into high- and low-adequate-LND-rate centers according to the median institution-level adequate LND rate (Supplementary Fig. 3). Overall survival did not differ between high- and low-adequate-LND-rate centers (log-rank *p* = 0.68; Supplementary Fig. 4).

### Prognostic Stratification by Pathologic Nodal Status According to Adequacy of LND

In an exploratory analysis evaluating the prognostic discrimination of pathologic nodal status according to the adequacy of LND, Nx was treated as N0. The C-index of pathologic nodal status for OS was higher among patients who underwent adequate LND than among individuals who did not undergo adequate LND (0.619, 95% CI 0.576–0.663 vs. 0.550, 95% CI 0.534–0.566). Similar findings were observed for RFS, with a higher C-index among patients who underwent adequate LND versus individuals who did not undergo adequate LND (0.611, 95% CI 0.571–0.650 vs. 0.535, 95% CI 0.520–0.549). These findings suggest that pathologic nodal status provided more informative postoperative prognostic stratification when adequate LND was performed.

## Discussion

Despite the central role of liver resection as curative-intent treatment for iCCA, postoperative recurrence affects 50–70% of patients, resulting in poor long-term outcomes and emphasizing the importance of refining surgical approaches.^1,17^ Among the various prognostic factors, lymph node metastasis is a well-established predictor of outcome, and several guidelines recommend evaluation of at least six lymph nodes for accurate staging.^1,4^ However, despite multiple prior studies, the prognostic impact of LND in iCCA remains uncertain.^5^ In this context, the current study was important, because it moved beyond the simple question of whether LND was beneficial and instead demonstrated that its prognostic relevance differed according to tumor burden. Using a large international multi-institutional database, a significant interaction was identified between TBS and adequate LND. Specifically, among patients with TBS ≥ 5.0, adequate LND was associated with better OS and RFS, whereas among patients with TBS < 5.0, adjusted OS and RFS did not differ according to adequate LND status. These findings were reproduced in the subgroup of clinically node-negative patients, and a similar interaction was observed between TBS and any LND. Importantly, the interaction between TBS and adequate LND remained even after additional adjustment for postoperative pathologic variables, supporting the robustness of the primary findings. Collectively, these data suggest that the prognostic relevance of LND in iCCA is heterogeneous and may depend on tumor burden.

Several prior studies and meta-analyses have investigated the role of LND for resectable iCCA; yet its therapeutic significance remains controversial.^18–21^ Zhou et al. reported that routine LND was not associated with prolonged OS and was instead linked to greater postoperative morbidity.^21^ Likewise, Ishii et al. noted no OS benefit of LND in the overall pooled cohort; however, in studies with a low risk of bias, LND was associated with superior OS (HR 0.76; 95% CI 0.59–0.98).^5^ One possible explanation for these discrepant findings is that the prognostic impact of LND may not be uniform across all patients. Rather, when evaluated across unselected cohorts, any prognostic association of LND may be diluted by inclusion of patients who are unlikely to derive oncologic benefit. LND is generally thought to serve at least two potential roles: first, more accurate nodal staging through identification of occult nodal disease, thereby improving postoperative prognostic assessment; and second, clearance of regional nodal disease.^18,22^ While enhancing prognostic classification, improved staging is unlikely to fully explain the observed differences in OS and RFS. As such, it is plausible that LND may have greater clinical relevance among patients with more advanced tumor burden and biologically aggressive disease. In the current study, this hypothesis was supported by the significant interaction between TBS and adequate LND, whereby the prognostic association of adequate LND was observed primarily among patients with TBS ≥ 5.0, but not among individuals with TBS < 5.0. A similar interaction was observed when any LND, rather than only adequate LND, was considered in the analysis. These findings may help to explain the discrepant results reported in the existing literature.

Tumor size and tumor number have long been recognized as important morphologic determinants of prognosis in iCCA.^23–25^ In addition, prior studies have suggested that larger tumors and multifocal disease are associated with a higher incidence of lymph node metastasis, implying that these preoperatively assessable morphologic features may reflect not only the anatomic extent of disease but also a more aggressive tumor phenotype with a greater propensity for regional spread.^18,26,27^ However, when tumor size and tumor number are considered separately, their interpretation often depends on arbitrary cutoffs and may fail to fully capture the overall extent of tumor burden. To address this limitation, Sasaki and colleagues developed the tumor burden score (TBS) as a single continuous metric integrating tumor size and number.^13^ Subsequent studies have demonstrated the prognostic utility of TBS in hepatic malignancies, including iCCA.^28–30^ Importantly, an Italian multicenter study by Sposito et al. further demonstrated that TBS also has value beyond prognostic stratification, as each 1-point increase in TBS was associated with a 9% increase in the odds of lymph node metastasis among patients undergoing adequate lymphadenectomy for iCCA.^31^ Taken together, these findings suggest that TBS may serve as a more comprehensive surrogate of oncologic burden than tumor size or tumor number alone. In this context, the favorable association of adequate LND with survival among patients with high TBS suggests that LND may have value not only for more accurate staging, but also as a component of more comprehensive regional disease management in patients with greater oncologic burden.

These findings also have important implications for current surgical practice. Although existing guidelines recommend evaluation of at least six lymph nodes in patients undergoing resection for iCCA, adequate LND remains inconsistently achieved in real-world practice.^13,32^ Recent studies have reported that guideline-concordant nodal evaluation is achieved in only 10–25% of patients.^20,32,33^ In the current cohort, adequate LND was likewise performed in only 20.7% of patients. In this context, the present data suggest that failure to achieve adequate nodal evaluation may be particularly consequential among patients with high TBS, in whom adequate LND was associated with improved OS and RFS. The current study underscores the importance of more consistent adherence to existing recommendations, especially in patients with greater preoperative tumor burden. From a practical standpoint, TBS may provide a simple preoperative framework to identify patients in whom a deliberate effort to achieve guideline-concordant lymphadenectomy is most likely to have oncologic relevance. Importantly, these findings should not be interpreted as evidence supporting omission of LND in patients with low TBS. Rather, the results suggest that the prognostic consequences of failing to achieve guideline-concordant nodal evaluation may be particularly pronounced among patients with high tumor burden.

Several limitations should be acknowledged. Despite the use of a large international multi-institutional database, inter-institutional variation in patient selection, surgical indications, and operative technique was unavoidable. The indication for LND and the extent of nodal dissection were likely influenced by surgeon- and center-specific practice patterns, which may have introduced residual selection bias. Particularly, although the institution-level sensitivity analysis did not demonstrate a survival difference between high- and low-adequate-LND-rate centers, residual institutional differences in multidisciplinary care, postoperative surveillance, recurrence-directed treatment, patient fitness for subsequent therapy, access to advanced systemic therapies, and genomic profiling could not be fully accounted for. In addition, pathologic nodal assessment was not standardized across centers, and differences in lymph node retrieval and examination may have influenced both TLNE and the detection of nodal metastasis. The relatively long study period, which was necessary given the rarity of iCCA, may also have introduced temporal heterogeneity in staging, surgical management, and perioperative care. Furthermore, the TBS cutoff identified in the study requires external validation before clinical implementation. Finally, because of the retrospective nature of the study, causal inferences cannot be made. Prospective studies with standardized surgical and pathologic protocols are needed to confirm the observed interaction between TBS and the prognostic impact of adequate LND.

## Conclusions

The prognostic relevance of adequate LND in patients undergoing curative-intent resection for iCCA was modified by tumor burden. Adequate LND was associated with improved OS and RFS among patients with high TBS, but not among individuals with low TBS. These findings indicate that the oncologic importance of guideline-concordant nodal evaluation may be greatest in patients with greater preoperative tumor burden. Accordingly, TBS may serve as a practical preoperative framework for contextualizing the relevance of LND, although prospective validation is warranted before clinical implementation.

## Supplementary Information

Below is the link to the electronic supplementary material.

Supplementary file1 (DOCX 2930 kb)

## Data Availability

Study data are not publicly available as they contain patient-level personal information but are available from the corresponding author on reasonable request.
