# Supplementary material for: Tumor Burden Modifies the Prognostic Impact of Adequate Lymph Node Dissection in Patients Undergoing Curative-Intent Resection for Intrahepatic Cholangiocarcinoma
Source: Ann Surg Oncol. 2026 Jun 14;33(9):7859–69. doi: 10.1245/s10434-026-19995-2 (PMC13452779; doi:10.1245/s10434-026-19995-2)
Supplement: Supplementary file 1 [file 10434_2026_19995_MOESM1_ESM.docx]

**Supplementary Table 1**. Multivariable COX regression analysis for overall survival, including an interaction term between lymph node dissection and tumor burden score.

|  | Multivariate analysis | |
| --- | --- | --- |
| Variables | HR 95%CI | *P* value |
| ASA classification, > 2 |  |  |
| Classification 1,2 | Ref |  |
| Classification, > 2 | 1.19 [1.03, 1.38] | **0.017** |
| Jaundice |  |  |
| No | Ref |  |
| Yes | 1.51 [1.19, 1.92] | **0.001** |
| Preoperative LN assessment |  |  |
| Negative | Ref |  |
| Positive | 1.23 [1.03, 1.46] | **0.022** |
| Major vascular invasion |  |  |
| No | Ref |  |
| Yes | 1.49 [1.22, 1.82] | **<0.001** |
| TBS * LND | 0.90 [0.86, 0.94] | **<0.001** |

**ASA,** American society of Anesthesiologists; **LN,** lymph node; **TBS,** tumor burden score; **LND,** lymph node dissection; Bold font signify *P* value < 0.05

**Supplementary Table 2**. Multivariable COX regression analysis for overall survival, including postoperative variables.

|  | Multivariate analysis | |
| --- | --- | --- |
| Variables | HR 95%CI | *P* value |
| ASA classification, > 2 |  |  |
| Classification 1,2 | Ref |  |
| Classification, > 2 | 1.32 [1.14, 1.54] | **<0.001** |
| Jaundice |  |  |
| No | Ref |  |
| Yes | 1.42 [1.11, 1.81] | **0.005** |
| Major vascular invasion |  |  |
| No | Ref |  |
| Yes | 1.10 [0.89, 1.36] | 0.389 |
| Margin |  |  |
| R0 | Ref |  |
| R1 | 1.12 [0.91, 1.36] | 0.285 |
| Pathological T category |  |  |
| T1/2 | Ref |  |
| T3/4 | 1.22 [1.03, 1.45] | **0.023** |
| Pathological N category |  |  |
| N0/Nx | Ref |  |
| N1 | 2.19 [1.81, 2.65] | **<0.001** |
| Microvascular invasion |  |  |
| No | Ref |  |
| Yes | 1.15 [0.97, 1.38] | 0.112 |
| Morphologic type |  |  |
| MF/IG | Ref |  |
| PI/MF+PI | 1.34 [1.09, 1.64] | **0.005** |
| Grade |  |  |
| well/moderate | Ref |  |
| poor/undifferentiated, | 1.18 [0.98, 1.41] | 0.080 |
| Perineural invasion |  |  |
| No | Ref |  |
| Yes | 1.23 [1.01, 1.50] | **0.041** |
| Adjuvant chemotherapy |  |  |
| No | Ref |  |
| Yes | 0.66 [0.56, 0.78] | **<0.001** |
| TBS * adequate LND | 0.90 [0.86, 0.94] | **<0.001** |

**ASA,** American society of Anesthesiologists; **LN,** lymph node; **MF,** mass-forming; **IG,** intraductal growth; **TBS,** tumor burden score; **LND,** lymph node dissection; Bold font signify *P* value < 0.05

**Supplementary Figure 1.** Adjusted overall survival by adequate lymph node dissection according to tumor burden score strata among patients with clinically node-negative.

**Supplementary Figure 2.** Adjusted recurrence-free survival by adequate lymph node dissection according to tumor burden score strata among patients with clinically node-negative.

**Supplementary Figure 3.** The distribution of institution-level adequate lymph node dissection (LND) rates.

**Supplementary Figure 4.** Kaplan–Meier analysis comparing overall survival between high- and low-adequate lymph node dissection (LND) rate centers.
